# Human Direct Actions May Alter Animal Welfare, a Study on Horses (*Equus caballus*)

**DOI:** 10.1371/journal.pone.0010257

**Published:** 2010-04-28

**Authors:** Clémence Lesimple, Carole Fureix, Hervé Menguy, Martine Hausberger

**Affiliations:** 1 Université de Rennes 1, Laboratoire d'éthologie animale et humaine EthoS - UMR CNRS 6552, Station Biologique, Paimpont, France; 2 Université de Rennes 1, Laboratoire d'éthologie animale et humaine EthoS - UMR CNRS 6552, Campus de Beaulieu, Rennes, France; 3 Chiropractic practice, St. Jacques de la Lande, France; L'université Pierre et Marie Curie, France

## Abstract

**Background:**

Back pain is the cause of bad welfare in humans and animals. Although vertebral problems are regularly reported on riding horses, these problems are not always identified nor noticed enough to prevent these horses to be used for work.

**Methodology/Principal Findings:**

Nineteen horses from two riding centres were submitted to chiropractic examinations performed by an experienced chiropractor and both horses' and riders' postures were observed during a riding lesson. The results show that 74% of horses were severely affected by vertebral problems, while only 26% were mildly or not affected. The degree of vertebral problems identified at rest was statistically correlated with horses' attitudes at work (neck height and curve), and horses' attitudes at work were clearly correlated with riders' positions. Clear differences appeared between schools concerning both riders' and horses' postures, and the analysis of the teachers' speech content and duration highlighted differences in the attention devoted to the riders' position.

**Conclusion/Significance:**

These findings are to our knowledge the first to underline the impact of riding on horses' back problems and the importance of teaching proper balance to beginner riders in order to increase animals' welfare.

## Introduction

In humans, both psychological (*e.g* [1]) and physical constraints at work may lead to chronic back pain [2]. Postural problems appear amongst the primary causes involved [3], [4]. Horses share with humans both to have a working activity that may involve physiological and physical stress [5] and a high prevalence of back pain problems [6]–[8]. In addition, because the expression of pain in this species may be low and in any case underestimated by owners [9]–[11] most horses keep being used for riding despite discomfort or pain. Apart from cases with overt associated lameness, horses mainly express these problems through progressive or sudden changes in temperament [11], leading to increased aggressiveness towards humans [12] or signs of escape attempts (*e.g.* [13], [5], [14]). Veterinarians, especially those involved in spine research, have long evoked work as a possible source or correlate of back pain in horses. Thus [15], [6], [8], [16], found differences in the prevalence, type and localization of spine disorders according to the type of work performed by the horse. According to Haussler [10] and Cauvin [11], improper riding techniques have to be identified as a potential source of back problems. For Ridgway & Harman [13], “equitation that produces physical or emotional stress must be identified or corrected” as otherwise treatments efforts may well be in vain.

Despite these clinical observations, little attention was given to the impact of work (*i.e.* riding) on horse welfare, which appears as a potentially underestimated problem [17], [14]. However, growing evidence is shown of physical and emotional stress associated with work in this species, leading to chronic effects. In a large scale study based on behavioural tests outside the working situation, [18] found that show horses, and especially dressage horses, exhibited higher emotional levels than unbroken or leisure horses. More recently, it was shown that the type and prevalence of abnormal behaviours performed in the box differed according to the type of work [19]: dressage horses in particular, exhibited more headshaking, which was suggested to be related to the stronger bit pressure at work that may damage this region of the mandible [20]. Horses with mouth pain tend to avoid it by raising the head, which causes extension of the back [13]. High neck posture associated with raised head has been shown to be the most uncomfortable posture for horses, affecting motion [21], [22]. Changes in head and neck positions significantly affected thoracolumbar kinematics in the unridden horses studied in these two reports (see also [8], [23]). If riding techniques affect neck and head position, they may therefore repeatedly affect the thoracolumbar system and lead to potential chronic back problems. However no study has been performed yet on a precise analysis of specific riders' aids on the horse's posture during riding [24].

In the present study, we focused on riding center horses that are confronted to unskilled riders. We hypothesized that in the “beginners”' lessons, undesirable hands or legs actions may impact on escape responses from horses [5] and lead to altered postures at work with potential chronic consequences. In a recent study, some riding center horses appeared to be too stiff to obtain a cervical flexion at work [25]. The present study combined precise observations of horses' and beginner riders' postures at work and analysis of their potential correlates with examination of the horses' spine by a licensed practitioner in the box. As we hypothesized that work was, through postural reactions, the source of potential back problems rather than their consequence, we analyzed how the riders' techniques were monitored by the riding teacher. The results show that teaching practices differed between the two riding centers studied, reflected by differences in the riders' postures that also obviously led to different postures of horses at work. Postures at work were clearly correlated with back pain problems outside work, supporting the hypothesis that stress at work may be responsible for chronic vertebral problems in horses.

## Methods

Experiments complied with the current French laws (Centre National de la Recherche Scientifique) related to animal experimentation and were in accordance to the European directive 86/609/CEE. Only behavioural observations and non painful examination were performed, as the chiropractic procedure is based on non painful (in the hands of a skilled manipulator) *e.g* [26], which was confirmed by the absence of any retreat behaviour of the horses. Animal husbandry and care were under management of the riding schools staffs, as this experiment involved horses from the field (no laboratory animals).

All the observed riders gave us their oral consent to be involved in the study, and a written consent of the riding teachers was obtained in each case. Riding teachers are, according to French laws, empowered to take this kind of decisions. Only behavioural observations were performed, and neither the riders nor the teachers were submitted to any other experimentation.

### Animals

The 19 tested horses (11 geldings, 8 mares; 7–22 years old; 8 breeds) were distributed across two riding centers (SA and SB) with similar activities and housing conditions **(** **Table 1** **)**. In all cases, the horses were kept singly in 3m * 3m straw-bedded individual boxes cleaned once a day. Animals were fed industrial pellets 3 times a day and hay once a day. Each box was equipped with an automatic drinker. Horses worked in riding lessons involving children and teenagers for 4–12 hours per week (with 1 closing day).

**Table 1:** Distribution of horses between schools.

	Number	Breeds							Ages
	of horses	SF	CO	UNR	TF	CHSL	PFS	PS	( 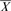 ± se)
SA	9	3	1	3	0	0	1	1	13.5±0.9
SB	10	5	0	3	1	1	0	0	15.1±1.2

SF: French Saddlebred, CO: Connemara, UNR: Unregistered horse, TF: French Trotter, CHSL: Saddle horse, PFS: French Pony, PS: Thoroughbred.

### Horses' spine examination

Although all authors agree that horse back problems are highly frequent, most also agree that their evaluation is difficult [6], [27], [28]. Radiographic imaging is limited by the thickness of the surrounding soft tissues [11]; ultrasonic, scintigraphic approaches all have an interest but remain difficult to apply in field conditions and on a large sample of horses [16], [11]. Studying kinematics of the spine requires fixed markers and horses in controlled conditions moving in front of fixed cameras (e.g. [29], [30], [7]). It was therefore not applicable here.

Chiropractic approach clearly addresses subclinical conditions (of special interest here) and licensed professionals have an expertise in the evaluation of joints and spinal related disorders [10], [9]. Therefore evaluation of our study horses' spine was performed by a 20 years experienced licensed chiropractor (H. Menguy) who was totally blind to the results of the observations performed during riding sessions and did not know the horses beforehand. Manual palpation was performed from head to tail. Manual methods have been suggested to be efficient to detect back pain ([31], [32].

In order to ensure the repeatability of these findings, evaluation was double performed by a second licensed 3.69 agreement, therefore confirming reliability of the evaluation.

Examination was performed in each horse's box outside working hours. The horse was slightly restrained by an unknown experimenter (M. Hausberger) who was also blind to the other data.

Horses were classified as totally exempt; slightly affected (often one vertebra affected) or severely affected as evaluated by the practitioner.

Data also included number of vertebrae affected and number of areas (*e.g.* cervical, thoracic, lumbar, sacral, coccygeal).

All the chiropractic evaluations were perform for free by H. Menguy himself, manager and only employee of the chiropractic practice. Moreover the manual palpations were carried on Sunday, outside working time of the practice.

### Measurements of horses and riders' postures

Two “beginners” (less than 50 hours practice) lessons were video-recorded using a JVC, Everio GZ-MG275 camcorder, which was on a tripod at a fixed place on the ground within the covered area used for lessons. Horses walked mostly along the wall on a pathway and the position of the camcorder enabled to film in a perpendicular position each horse-rider pair every time they crossed the camcorder “field of vision”. The camcorder was at a distance of 25 m from the pathway. Only postures during walk were retained as it allowed more precise observations (slower pace) and more homogenous data (less impact on riders of horses morphology, riders always sitting).

A scan sampling approach was used. The postures of both horse and rider were measured at the precise moment they were in the centre of the camcorder image.

In average, 10.74±1.04 scans were obtained for each pair. Given the growing evidence of a major impact of the neck position on the kinematics of the thoracolumbar spine in horses (e.g. [22], [21]) in accordance with the “bow-string” theory for ungulates (Strasser 1913 cited by [6]), we focused on the horses' neck height and shape. In particular mouth escape responses involve high and hollow neck [13].

**Figure 1:**
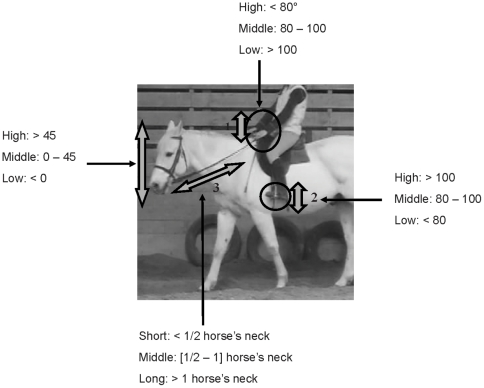
Horses' and riders' different postures at work.

Horses' neck evaluations therefore involved **(** **Fig 1** **)**:

- Height: horizontal (0°–45°/back line), high (>45°/back line) and low (<0°/back line).
- Shapes: round (convex), flat (no curve) and hollow (concave).

Observations of riders postures focused on hands and legs actions, considered as the most prone to induce potential stress ([5]) **(** **Fig 1** **)**:

- Hands height: high (elbow angle <80°), middle (elbow angle [80°–100°]) and low (elbow angle >100°).
- Heels height: high (ankle angle >100°), middle (ankle angle [80°–100°]), low (ankle angle <80°).

Reins' length was also evaluated as it may determine a softer (longer reins) or harder (shorter reins) contact with the horse's mouth. Reins lengths were categorized in short (less than half the horse's neck length), medium (from half to the horse's neck length) and long (more than the horse's neck length).

### Assessment of teaching practice

As beginners do not master totally seat balance and aids (legs, hands) actions, it seemed probable that their teachers' advices played a major role at that stage in “shaping” riders postures. Therefore we analyzed the riding teachers' speech during lessons in order to evaluate if 1) they were active during lessons, 2) they were strongly or not monitoring the riders' postures, 3) when they were doing that, what parts of the riders' actions they were paying most attention to.

Their speech was recorded continuously using a digital voice recorder (Thomson DK 300). Further analysis involved 1) total speech duration, 2) number of speech bouts, 3) number of mentions of the riders' posture within bouts, 4) type of mentions.

Data correspond to 1 hour continuous recording.

### Data and statistical analysis

Data collected in relation with the spine's state were nominal variables (*i.e.* fully exempt/slightly affected/severely affected) and percentage of affected vertebras/vertebrae per area. Data collected in relation with horses and riders were percentage of lesson time spent in each position. As data were not normally distributed, we used non-parametric statistical tests [33]. Spearman correlation tests were used to detect existing links between riders' position, horses' attitude at work and horses' vertebrae problems. Chi square and Kruskall-Wallis tests were used to compare horses' vertebral state, riders' position and teachers' speech. Mann-Whitney *U* -tests were used to assess possible differences in horses' attitude and riders' position at work between schools. These analyses were conducted using Statistica© 7.1 software (accepted p level at 0.05).

A more descriptive, but very interesting approach consists of using a factorial correspondence analysis. Each factor is represented in a plan with 2 axes. These axes can be interpreted by considering the factor loadings of initial variables, which means squared correlation coefficient between each variable and each axis. Data used were the frequencies of each observed posture, for the horse and for the rider. A homemade software, GTabm [34], was used.

## Results

### Horses' vertebral disorders and work postures

Evaluation of the spine state in the box revealed that, in accordance with previous studies (*e.g.* [8], [27], [35]) a large majority of horses had clear vertebral disorders (*N* = 14, 74%), while only 21% of the horses were evaluated as slightly affected and only one as totally exempt. About 60% of the horses were affected in more than one area.

The evaluated percentage of affected vertebrae per horse varied largely (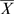 ± SE = 25±5.77, range: 0–88). No difference was found according to sex (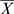
_♀_ ± SE = 30±6.53, 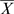
_♂_ ± SE = 22±8.87, Mann Whitney U test: *U* = 28, *N_♀_* = 8, *N_♂_* = 11, *P*>0.05) or age (Spearman correlation test, *r_s_* = −0.32, *N* = 19, *P*>0.05).

Horses spent on average 60% of the scans with an horizontal neck (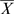 ± SE = 67.02±5.09), 10% with a high neck (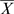 ± SE = 12.54±4.67) and 20% with a low neck (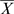 ± SE = 20.44±4.34).

They had mostly a round neck (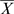 ± SE = 62.19±6.79% of scans), but flat (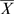 ± SE = 30.72±4.88% of scans) and hollow (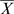 ± SE = 7.09±3.93% of scans) neck could also be observed. Out of the 19 horses, 10 were never observed with a high neck and 15 were never observed with a hollow neck.

Neck position and shape were correlated: a hollow shape was positively correlated with a high position (Spearman correlation test, *r_s_* = 0.66, *N* = 19, *P* = 0.002) and negatively with a horizontal position (Spearman correlation test, *r_s_* = −0.51, *N* = 19, *P* = 0.02).

Vertebral disorders evaluated in the box were correlated with postural elements during work. Thus, the number of vertebral areas affected was positively correlated with the time spent at work in a high neck position (Spearman correlation test, *r_s_* = 0.53, *N* = 19, *P* = 0.02). Less thoracic vertebrae were affected if the horse worked with a low neck posture (Spearman correlation test, *r_s_* = −0.60, *N* = 19, *P*<0.01).

Slightly or not affected horses were never observed with a high neck position contrarily to the severely affected horses (number of scans: 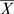
_slightly affected_ ± SE = 0±0, 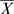
_severely affected_ ± SE = 17.01±5.92; Mann-Whitney U test: *U* = 10, *N_slightly affected_* = 4, *N_severely affected_* = 14 *P*<0.05). In addition, they spent more time in a low neck posture than the latter (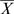
_slightly affected_ = 30.94±2.57, 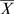
_severely affected_ = 18.91±5.48; Mann-Whitney U test: *U* = 9, *N_slightly affected_* = 4, *N_severely affected_* = 14, *P*<0.05).

### Riders' postures and correlates with horses

Large individual variations were observed but on average riders spent more time with low hands (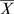 ± SE = 43.64±4.79% of scans, range 12.5–88.89; high hands: 25.91±4.93% of scans, range 0–77.78; middle hands: 30.45±4.45% of scans, range 0–60), middle heels (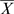 ± SE = 59.17±5.24% of scans, range 0–85.71; high heels: 16.22±3.52% of scans, range 0–55.55; low heels: 24.60±6.67% of scans, range 0–100). They tended to have mostly medium reins (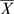 ± SE = 54.78±5.17% of scans, range 14.28–77.78; short: 1.5±1.03% of scans, range 0–14.28; long: 43.72±5.31% of scans, range 12.5–81.7).

Interestingly, riders with high heels also tended to spend more time with medium reins (Spearman correlation test, *r_s_* = 0.47, *N* = 19, *P*<0.05), and less with long reins (Spearman correlation test, *r_s_* = −0.54, *N* = 19, *P*<0.02).

Clear correlates appeared between riders' postures and horses' neck position **(** **Table 2** **)**.

**Table 2:** Correlations between riders' and horses' postures at work.

Rider	Low hands	High hands	Long reins	Medium reins	High heels	Low heels
Horse						
Low neck					rs = −0.46 p<0.05	rs = 0.51 p<0.05
Horizontal neck		rs = −0.53, p<0.05		rs = −0.51 p<0.05		
High neck	r_s_ = −0.60 p<0.01		rs = −0.53 p<0.05	rs = 0.59 p<0.01	rs = 0.50 p<0.05	
Round neck	rs = 0.58 p<0.01	r_s_ = 0.48 p<0.05			rs = −0.60, p<0.01	
Hollow neck	rs = −0.62 p<0.01		rs = −0.46 p<0.05			

Only the statistically significant correlations (Spearman correlation test) are presented here. All others were NS.

Hands positions were correlated with the horses' neck height and shape: the more the rider was with low hands, the more the horse exhibited a round neck shape (Spearman correlation test, r_s_ = 0.58, *N* = 19, *P*<0.02), and the less it was with high (Spearman correlation test, *r_s_* = −0.60, N = 19, *P*<0.01) and/or hollow (Spearman correlation test, *r_s_* = −0.62, *N* = 19, *P*<0.01) neck. On the contrary, the more time the rider spent with high hands, the more the horse was observed in a high neck position (Spearman correlation tests, *r_s_* = 0.48, *N* = 19, *P*<0.05) and the less with an horizontal (Spearman correlation tests, *r_s_* = −0.53, *N* = 19, *P*<0.05) neck position.

Reins length was also influential: the more time the rider spent with long reins, the less the horse was observed with a high (Spearman correlation test, *r_s_* = −0.53, *N* = 19, *P*<0.05) and/or hollow (Spearman correlation test, *r_s_* = −0.46, *N* = 19, *P*<0.05) neck. Medium reins correlated positively with horses' high neck occurrences (Spearman correlation test, *r_s_* = 0.59, *N* = 19, *P*<0.01) and negatively with horizontal height (Spearman correlation test, *N* = 19, *r_s_* = −0.51, *P*<0.05).

Finally, the more the rider had low heels, the more time the horse spent in a low neck position (Spearman test, *r_s_* = 0.51, N = 19, *P*<0.05).

### Comparison between riding schools

The evaluation of spine disorders at rest in the box revealed important differences between schools **(** **Table 3** **)**, with more vertebrae affected in S_A_ (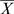 ± SE = 18.78±4.63) than in S_B_ (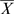 ± SE = 7.80±3.07) and more vertebral areas affected in S_A_ (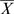 ± SE = 2.78±0.52) than in S_B_ (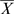 ± SE = 1.20±0.34) (Mann-Whitney U test: *U* = 16.5, *N_SA_* = 9, *N_SB_* = 10, *P*<0.05). All 4 slightly affected horses and the one totally exempt belonged to S_B_. In total, 50% of S_B_ horses were severely affected, while 100% were so in S_A_ (Fischer exact test, *P*<0.05).

**Table 3:** Horses' Postural and vertebral characteristics in the two riding schools.

	Neck						Vertebral problems			
	High	Horizontal	Low	Hollow	Flat	Round	Exempt/Slightly affected	Severely affected	Number of affected areas	Number of affected vertebrae
SA	24.99±8.09	61.76±8.45	13.25±5.58	14.51±7.74	38.84±7.06	46.66±10.75	0	9	2.77±0.52	18.78±4.63
SB	1.32±0.96	71.75±6.02	26.92±6.07	0.42±0.42	26.92±6.07	76.16±6.11	5	5	1.2±0.34	7.8±3.07

Differences also occurred at work, with more S_A_ horses observed at least once in high neck position (*χ* ^2^_1_: N_SA_ = 7, N_SB_ = 2, *P*<0.02). Moreover, S_A_ horses spent a larger proportion of time in a high neck posture than SB horses (% of time: 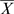
_SA_ ± SE = 24.99±8.09, 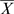
_SB_ ± SE = 1.32±0.96 Mann-Whitney *U* test: *U* = 12, *N_SA_* = 9, *N_SB_* = 10, *P*<0.005). In consequence, they also spent less time with a low neck posture (% of time: 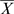
_SA_ ± SE = 13.25±5.58; 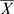
_SB_ ± SE = 26.92±6.08, Mann-Whitney *U* test: *U* = 20.5, *N_SA_* = 9, *N_SB_* = 10, *P*<0.05) **(** **Table 3** **)**.

**Figure 2:**
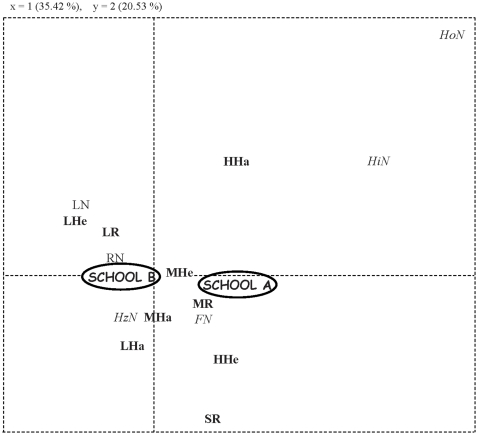
FCA results based on horses' and riders' postures at work. Riders' hands: high HHa, middle MHa, low LHa; Riders' heels: high HHe, middle MHe, low LHe; Reins length: short SR, medium MR, long LR, Horses' neck: high HN, horizontal HN, low LN, hollow HoN, flat FN, round RN.

Actually, clear differences in the global postural profiles appeared between riding schools. Thus a FCA performed on both riders and horses showed that the two first axes explained 56% of the variance **(** **Fig 2** **)**. Axis 1 opposes riders' short reins and horses' high and hollow neck to riders' low heels and horses low and round neck. Axis 2 opposes rider's low hands, short reins and high heels to horse's high and hollow neck **(** **Table 4** **)**.

**Table 4:** Factor loadings of the Factorial Correspondence Analysis.

	Factor loadings of variable		
Horses' Postures	F1	F2	F3
*High neck*	1395	525	50
*Horizontal neck*	−101	−211	−22
*Low neck*	−526	371	45
*Hollow neck*	1962	1145	−380
*Flat neck*	276	−216	−186
*Round neck*	−359	−24	134
Riders' Postures			
*High hands*	426	570	219
*Middle hands*	−2	−235	−338
*Low hands*	−253	−174	105
*High heels*	495	−441	237
*Middle heels*	135	−31	231
*Low heels*	−646	364	−708
*Short reins*	332	−686	1735
*Medium reins*	268	−119	−95
*Long reins*	−347	171	60
School			
*SA*	308	−53	68
*SB*	−277	46	−60

Axis 1 clearly separated both riding schools, with S_A_ showing mostly horses that had a high neck with a hollow or flat shape. These horses' postures were associated with riders having high heels and hands, and short or tight reins.

S2 horses on the contrary presented mostly horizontal or low neck with a round shape while riders tended to present low heels and hands as well as long reins **(see** **Table 3** **and** **Table 5** **)**.

**Table 5:** Riders' postural characteristics in the two riding schools.

	Hands			Reins Length			Heels		
	High	Middle	Low	Short	Medium	Long	High	Middle	Low
SA	33.57±7.94	25.16±5.95	41.27±8.62	3.17±2.10	62.66±7.04	34.17±7.04	26.45±4.84	64.86±5.17	8.68±5.98
SB	19.02±5.56	35.22±6.44	45.76±5.16	0±0	47.68±7.09	52.32±7.09	7.02±2.91	54.04±8.76	38.94±9.62

Postural profiles of horses and riders not only appeared to be related but clearly discriminated both riding schools, questioning the impact of teaching practices.

### Teaching practices

Teachers of both schools differed in their amount of speech during a riding lesson (S_A_: 2090 s, S_B_: 2506 s, Chi-square test: *χ* ^2^_1_ = 37.65, *P*<0.001) with more speech bouts initiated by S_A_ teacher (*N_SA_* = 74, *N_SB_* = 107, Chi-square test: *χ* ^2^_1_ = 6.35, *P*<0.05) **(** **Table 6** **)**.

**Table 6:** Characteristics of the riding teachers' speech in each riding school.

	Target				Hands position		Reins length		Legs position	
					(Nb of speech bouts)					
	Amount of speech (s)	Nb of speech bouts	Horses' position in the group	Riders' posture (% of speech bouts)	“Lower”	“Move hands forward”	Longer	Shorter	Tighten	Direction of the riders' gaze
SA	2090	74	14	81%	1	3	10	21	2	1
SB	2506	108	1	99%	8	15	32	4	15	14

In S_B_, the teacher devoted 99% of the speech bouts to the riders' posture and only 1% to the horses' position in the group, while a larger number (20%) was devoted to the control on horse in S_A_ (“You are too close”, meaning your horse is too close to the preceding one: *N_SA_* = 13, *N_SB_* = 1, Chi-square test, *χ* ^2^_1_ = 10.28, *P*<0.001) **(** **Table 6** **)**.

S_B_ teacher required riders to lower their hand (“Lower your hands”, “Keep your hands low”) and move them forward (“Move your hands forward”, meaning less rein tension) while this almost never happened with S_A_ teacher (lower hands: *N_SA_* = 1, *N_SB_* = 8, Chi-square test: *χ* ^2^_1_ = 5.44, *P*<0.05, “Move your hands forward”: N_SA_ = 3, N_SB_ = 15, Chi-square test: *χ* ^2^_1_ = 8, *P*<0.005).

Both teachers equally paid attention to rein length (*N_SA_* = 31, *N_SB_* = 36), but while S_B_ teacher asked more often for longer reins (“Extend your reins”) (*N_SA_* = 10, *N_SB_* = 32; Chi-square test: *χ* ^2^_1_ = 11.52, *P*<0.001), S_A_ teacher asked more for shorter reins (“Shorten your reins”, “Your reins are too long”) (*N_SA_* = 21, *N_SB_* = 4, Chi-square test, *χ* ^2^_1_ = 11.56, *P*<0.001) **(** **Table 6** **)**.

S_A_ teacher was more attentive to legs position, and asked more the riders to tighten their legs (*N_SA_* = 1, *N_SB_* = 13, Chi-square test, *χ* ^2^_1_ = 10.29, *P*<0.001). He also paid more attention to the direction of riders' gaze (*N_SA_* = 1, N_SB_ = 14, Chi-square test, *χ* ^2^_1_ = 11.27, *P*<0.001) **(** **Table 6** **)**.

Therefore, teachers' attention to the riders' posture and teaching strategies clearly differed between riding schools and were even opposite on aspects such as reins' length.

## Discussion

This study, based on riding school horses, is the very first to clearly demonstrate a relation between posture at work and vertebral problems evaluated at rest. Observations during work revealed that horses' and riders' postures were correlated while analysis of the teachers' speech to the riders strongly suggested that attention to the riders' postures may be determinant.

In the whole, this set of data inferred that improper riding postures may have a strong effect on horses' postures at work that may also lead to chronic vertebral problems. Comparisons of the two riding schools showed that there are “global profiles” with one case where the teacher was very attentive to riders' positions, riders had lower hands, and horses lower necks, while in the other case the teacher was more attentive to horse's control and riders tended to have higher hands and horses higher necks. Both centers differed also with the first having a much lower proportion of horses with vertebral problems than the latter.

Examination of the spine state revealed that most horses had back problems, actually all of them in one of the riding schools. This finding is in agreement with literature data: western horses were found to all have some thoracolumbar pain [8]; 78% of the 443 horses investigated by Jeffcott [15] had potential back pain (vertebral lesions and/or soft tissue injury); 92% of the dead race horses studied by Haussler *et al* [35] had thoracic impingements independently of their age. For Jeffcott [36], back pain in horses is one of the most common and least understood problems in sporting horses. Both Fonseca *et al* [8] and Jeffcott [15] found differences in the prevalence and type of vertebral problems according to the type of work performed. These observations add to the propositions of varied clinicians that riding techniques may be one of the potential sources of back problems [33], [11], [13].

In the present study, we found that severely affected horses were also those that spent more of their working time with a high and sometimes hollow neck. While this could be a consequence of their back problems, extending their spine in order to try and escape the potential pain due to the rider's additional weight, data from the “rider's side” suggest that horses' postures at work may rather be a consequence of riders' technique (or in the present case, lack of technique).

High neck postures are often observed when horses react to undesirable bit actions [5], [20], [13], which may have been the case here as beginners may have less control on their hands and having them high may have, through further muscular tension, increased this lack of control, and therefore the repeated actions of the bit on the horse's mouth. This particularly happened in the riding school where, under the teacher's demand, riders also tended to have shorter reins. They also had higher heels, which revealed tension and unbalance.

Neck position affects the thoracocolumbar system [21], [22], [8], [23]. Repeated undesirable postures at work may therefore lead to chronic damages of the spine [13], as observed in humans [4], [3]. The strong correlations found between riders' and horses' postures on the one hand, horses' postures at work and chronic vertebral problems on the other hand, are especially remarkable as observations relied upon a limited amount of working time. This suggests that these are strong effects that are particularly influential when repeated up to several hours a day.

This study is one of the rare ones to investigate the impact of riding per se on the horse's welfare (see also [14]). Impact of work on back pain is well known in humans but has been largely underestimated in horses [17]. Recent studies suggest that riding may impinge on chronic states, potentially leading to increased emotionality [18] or stereotypies [19]. The present study supports the idea that riding techniques may induce a chronically altered welfare. Only a precise analysis of riders' aids and their relation to horse postures could lead to such findings (see also [24]).

These results add to the range of factors that have to be taken into account when studying welfare. This questioning may extend to all species that work with humans were devices could induce undesirable postures (camels, donkeys…).

It is interesting to note that riding teachers can greatly differ in the attention they devote to their riders' postures. Unawareness of the link between riders' actions and horses' spine kinematics is a problem that the lack of scientific data could not help improve. The present findings have clear applied implications by promoting more awareness of the impact of human direct actions, leading to more attention to teach proper balance to beginner riders.

Chronic discomfort due to vertebral problems has been shown to increase horses' aggressiveness towards humans (Fureix *et al* in revision), reminding that human and animal welfare are linked when domestic animals are concerned.
