# Correction: Human Direct Actions May Alter Animal Welfare, a Study on Horses (*Equus caballus*)

**DOI:** 10.1371/annotation/530e1439-fee8-434c-97b1-7d2cd87e2e46

**Published:** 2010-05-24

**Authors:** Clémence Lesimple, Carole Fureix, Hervé Menguy, Martine Hausberger

The following paragraph in the Methods subsection "Horses' spine examination" should read as follows: "In order to ensure the repeatability of these findings, evaluation was double performed by a second licensed chiropractor, equally blind to results and unknown to horses, on 10 out of the 19 horses. Comparison of evaluations (localisation of vertebral sites affected) revealed a 94.28% ± 3.69% agreement, therefore confirming reliability of the evaluation."

